# Double Steal Phenomenon: Emergency Department Management of Recurrent Transient Ischemic Attack

**DOI:** 10.5811/cpcem.2019.1.40960

**Published:** 2019-02-13

**Authors:** Amit R. Rawal, Collin Bufano, Omar Saeed, Asif A. Khan

**Affiliations:** * North Florida Regional Medical Center, Department of Emergency Medicine, Gainesville, Florida; † University of Tennessee Health Science Center, Department of Internal Medicine, Memphis, Tennessee; ‡ North Florida Regional Medical Center, Department of Vascular and Interventional Neurology, Gainesville, Florida

## Abstract

Double steal phenomenon is a rare condition where occlusion of the innominate (brachiocephalic) artery leads to hemodynamic changes in which blood flow is shunted from the intracranial circulation down the right vertebral artery and subsequently up the right carotid and subclavian circulation. This is a case of a 67-year-old female presenting emergently with recurrent transient ischemic attacks due to double steal phenomenon. Emergency department recognition of the double steal phenomenon and large vessel occlusion by computed tomography angiogram of the head and neck allowed for early treatment, which was critical in avoiding irreversible cerebral infarction.

## INTRODUCTION

Subclavian steal syndrome is a well-documented phenomenon in which right vertebral artery flow is reversed due to a prevertebral stenosis of the subclavian artery. This is contrasted by double subclavian steal phenomenon, which is considered even rarer and more critical, and occurs secondary to stenosis of the brachiocephalic (innominate) artery. This results in blood being “stolen” from the intracranial basilar artery circulation down the right vertebral artery, reconstituting in the right subclavian artery, and then the flow is subsequently again “stolen” from the right subclavian artery in a retrograde fashion up the right carotid artery. This is a case of a 67-year-old female who arrived to the emergency department (ED) with recurrent transient ischemic attacks (TIA) exacerbated by right arm exercise and change from supine positioning. The etiology was found to be innominate artery occlusion and resultant double steal phenomenon.

## CASE REPORT

A 67-year-old female with history of chronic tobacco use, chronic obstructive pulmonary disease, hypertension, and hyperlipidemia, presented to the ED with symptoms of TIA. The patient described the acute onset of left-sided facial weakness that waxed and waned, recurring several times throughout the day, and lasting 2–3 minutes at a time. The left facial weakness was also associated with mild, left-arm weakness and “clumsiness” involving fine motor function of her left hand. She noted lightheadedness but denied leg weakness, headache, visual changes, chest pain or shortness of breath. She also noted that symptoms were brought on by use of her upper extremities and when she changed her body position from lying to sitting. She denied any similar symptoms previously or stroke history. Of note, she noticed a rapid improvement in her symptoms to resolution just prior to ED presentation.

On examination, her blood pressure (BP) was 183/86 millimeters of mercury (mmHg). She was awake, alert, oriented, and able to describe a detailed history. Her cranial nerves were intact, motor strength was 5/5 bilaterally, and fine motor movements in both her hands were normal. There was no ataxia, extraocular muscle dysfunction, or indication of posterior circulation involvement.

**Image 1:**
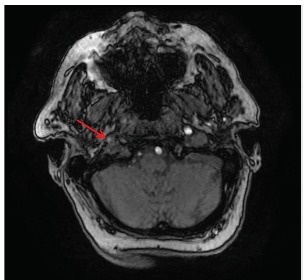
Computed tomography angiography of the neck showing a calcified flow restricting lesion (red arrow) at the origin of the innominate artery.

**Image 2:**
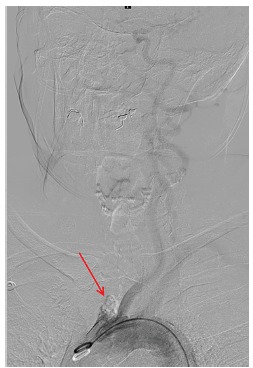
Magnetic resonance angiography of the head showing decreased contrast intensity in the right carotid artery (red arrow) compared to the left.

Just after her initial asymptomatic presentation to the ED, her symptoms recurred when her systolic BP dropped by 20 mmHg upon standing from a supine position. Emergent computed tomography angiogram (CTA) of the head and neck demonstrated a severe flow-limiting lesion of the innominate artery (Image 1). Further investigation with magnetic resonance imaging demonstrated decreased signal intensity within the right internal carotid artery at the cavernous sinus and petrous segments, a finding that potentially represented slow flow (Image 2).

**Image 3:**
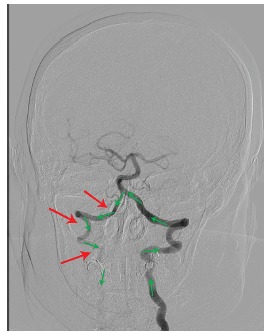
Aortic arch angiography demonstrated occlusion of the brachiocephalic artery (red arrow).

**Image 4:**
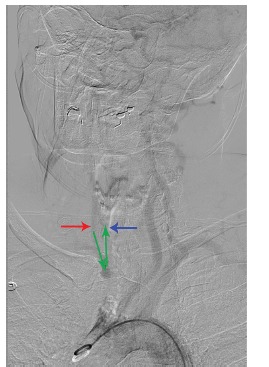
Percutaneous angiography demonstrating contrast, and thus perfusion (green arrows), being stolen from the left vertebral artery to the right vertebral artery (red arrows).

**Image 5:**
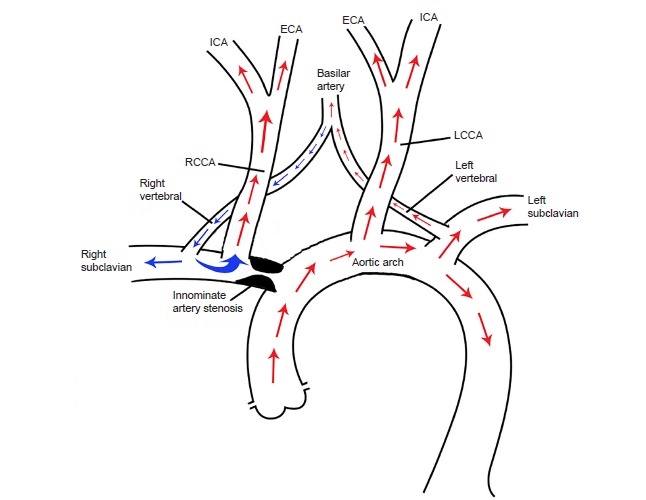
Percutaneous angiography demonstrating reconstitution of the brachiocephalic artery distal to the occlusion with secondary steal of perfusion (green arrows) from the vertebral artery (red arrow) up the right common carotid (blue arrow).

The patient subsequently underwent emergent cerebral angiogram, which demonstrated occlusion of the proximal innominate artery (Image 3) at the aortic arch with resultant left to right vertebral artery steal phenomenon supplying the right subclavian artery (Image 4). The distal brachiocephalic artery flow was reconstituted via the subclavian artery and secondary steal phenomenon occurred into the right common carotid artery, causing delayed flow to the right cerebral hemisphere (Image 5).

**CPC-EM Capsule**

What do we already know about this clinical entity?

*Double subclavian steal syndrome is a result of an occlusion in the innominate artery and causes hemodynamic flow changes in the right vertebral and carotid arteries, leading to neurologic deficits*.

What makes this presentation of disease reportable?

*This case was unique due to the recurrence of focal neurologic deficits that were precipitated by arm movement, blood pressure changes, and position changes*.

What is the major learning point?

*Computed tomography angiogram (CTA) of head and neck should be considered in the emergency department (ED) for evaluation of patients with neurologic symptoms that are recurrent, positional, or recur with labile pressures*.

How might this improve emergency medicine practice?

*This will hopefully encourage ED practitioners to have a low threshold for CTA on a subtype of neurologic patients to hasten diagnosis and prevent further ischemic events*.

The patient was maintained on a norepinephrine bitartrate infusion to increase BP, and her symptoms subsequently resolved. The symptoms recurred when she was positioned supine, but upon being placed in the Trendelenburg position her symptoms again resolved. The patient was therefore maintained with systolic BP goals between 160 and 210 mmHg. She remained asymptomatic during this period of elevated BP management. For definitive care, she underwent elective left carotid to right carotid “necklace” bypass surgery with complete and permanent resolution of her symptoms.

## DISCUSSION

Double steal phenomenon is caused by occlusion of the innominate artery. This results in hypoperfusion of the right carotid, subclavian and vertebral circulation, putting patients at risk of both posterior and anterior circulation ischemic events (Figure).^1^,^2^ Therefore, early diagnosis with CTA is critical. An ED evaluation of a subtype of TIAs (recurrent TIAs, positional TIAs, and TIAs that recur with labile blood pressures) to include CTA of the head and neck is warranted to identify lesions requiring emergent management.^3^ In this case, the double steal-inducing lesion at the origin of the brachiocephalic trunk resulted in weakness of the left face, hand and arm, as well as presyncope and dizziness.^4^

One of the more interesting aspects of the presentation was the positional nature of the symptoms, which resolved with induced hypertension. Additional signs and symptoms of innominate artery disease depend on degree of stenosis and acute changes in BP. Severe to near-total occlusion can cause additional symptoms that can originate from the vertebrobasilar circulation (vertigo, ataxia, drop attack, diplopia, and blurred vision), to symptoms that are hemispheric in origin (amaurosis fugax, transient paresis, and even upper extremity ischemia).^5^

## CONCLUSION

Symptomatic patients with double steal phenomenon can present with persistent focal neurological deficits, TIAs, recurrent syncopal episodes, and dizziness. Definitive diagnosis occurs with CT and cerebral angiography. This case highlights the importance of early recognition in an ED setting with timely goals to include permissive hypertension or induced hypertension until definitive surgical repair can be performed.^6^

## Figures and Tables

**Figure:**
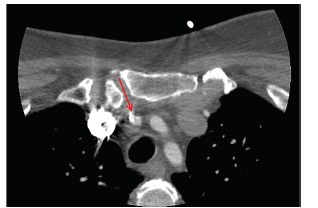
Schematic diagram demonstrating double steal phenomenon with retrograde blood flow from right vertebral artery to both right subclavian artery and the right common carotid artery (blue arrows). *RCCA*, right common carotid artery; *ICA*, internal carotid artery; *ECA*, external carotid artery; *LCCA*, left common carotid artery.
